# The genome sequence of the Ruddy Darter, *Sympetrum sanguineum *(Müller, 1764)

**DOI:** 10.12688/wellcomeopenres.23466.1

**Published:** 2025-01-15

**Authors:** Liam M. Crowley, Denise C. Wawman, University of Oxford and Wytham Woods Genome Acquisition Lab, Darwin Tree of Life Barcoding collective, Wellcome Sanger Institute Tree of Life Management, Samples and Laboratory team, Wellcome Sanger Institute Scientific Operations: Sequencing Operations, Wellcome Sanger Institute Tree of Life Core Informatics team, Tree of Life Core Informatics collective, Darwin Tree of Life Consortium

**Affiliations:** 1 University of Oxford, Oxford, England, UK

**Keywords:** Sympetrum sanguineum, Ruddy Darter, genome sequence, chromosomal, Odonata

## Abstract

We present a genome assembly from a male specimen of *Sympetrum sanguineum* (Ruddy Darter; Arthropoda; Insecta; Odonata; Libellulidae). The haplotype-resolved assembly contains two haplotypes with total lengths of 1,500.53 megabases and 1,304.05 megabases. Most of haplotype 1 is scaffolded into 13 chromosomal pseudomolecules, including the X sex chromosome, while haplotype 2 is scaffolded into 12 autosomes.

## Species taxonomy

Eukaryota; Opisthokonta; Metazoa; Eumetazoa; Bilateria; Protostomia; Ecdysozoa; Panarthropoda; Arthropoda; Mandibulata; Pancrustacea; Hexapoda; Insecta; Dicondylia; Pterygota; Palaeoptera; Odonata; Epiprocta; Anisoptera; Cavilabiata; Libellulidae; *Sympetrum*; *Sympetrum sanguineum* (Müller, 1764) (NCBI:txid)

## Background

The Ruddy Darter *Sympetrum sanguineum* is a small dragonfly in the family Libellulidae. It is 20–26 mm long with a hind wing measuring 23–29 mm. Mature males have a blood-red abdomen with darker sides and females are yellowish but may turn red as they age. Both sexes have a black line running down their sides from the frons to the hind part of the abdomen. Unlike other darters, they have entirely black legs, and unlike the Common Darter *Sympetrum striolatum* which has a straight abdomen, *S. sanguineum* has a waisted appearance ( Brooks, 2007).

*Sympetrum sanguineum* is most common around shallow lakes, ponds, ditches and canals, that are well vegetated. Although it is found throughout Ireland, in the United Kingdom it is mostly found in the south of England, with migrants from Continental Europe boosting the population. It flies from late June to October. The larvae feed on the roots of plants, including bulrushes *Typha* spp. and horsetails *Equisetum* spp., usually completing their lifecycle in one year ( Brooks, 2007).

The flight of *S. sanguineum* has been investigated extensively ( Wakeling & Ellington, 1997a; Wakeling & Ellington, 1997b; Wakeling & Ellington, 1997c), and some of the information gained has been used to help with the development of types of miniature flying robots known as flapping-wing micro aerial vehicles ( Szabo & D’Eleuterio, 2018).

We present a chromosomally complete genome sequence for *Sympetrum sanguineum* based on one adult male specimen collected at Wytham Woods, Oxfordshire, UK as part of the Darwin Tree of Life Project. This project is a collaborative effort to sequence all named eukaryotic species in the Atlantic Archipelago of Britain and Ireland ( Blaxter *et al.*, 2022).

## Genome sequence report

**Figure 1.**
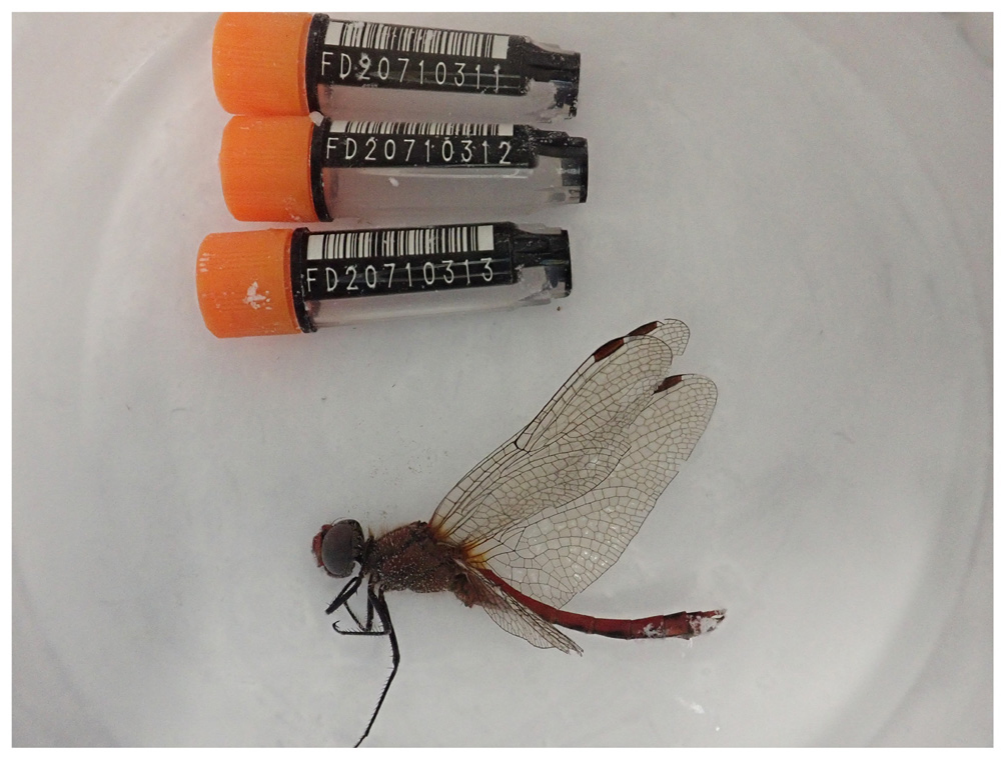
Photograph of the *Sympetrum sanguineum* (ioSymSang1) specimen used for genome sequencing.

The genome of an adult male *Sympetrum sanguineum* ( Figure 1) was sequenced using Pacific Biosciences single-molecule HiFi long reads, generating a total of 25.47 Gb (gigabases) from 2.11 million reads, providing approximately 23-fold coverage. Primary assembly contigs were scaffolded with chromosome conformation Hi-C data, which produced 109.20 Gb from 723.20 million reads. Specimen and sequencing details are provided in Table 1.

**Table 1.** Specimen and sequencing data for *Sympetrum sanguineum*.

Project information			
**Study title**	Sympetrum sanguineum (ruddy darter)		
**Umbrella BioProject**	PRJEB60309		
**Species**	*Sympetrum sanguineum*		
**BioSample**	SAMEA110451513		
**NCBI taxonomy ID**	197176		
Specimen information			
**Technology**	**ToLID**	**BioSample accession**	**Organism part**
**PacBio long read sequencing**	ioSymSang1	SAMEA110451696	thorax
**Hi-C sequencing**	ioSymSang1	SAMEA110451696	thorax
Sequencing information			
**Platform**	**Run accession**	**Read count**	**Base count (Gb)**
**Illumina NovaSeq 6000 (Hi-C)**	ERR12737259	1.58e+09	238.79
**Illumina NovaSeq 6000 (Hi-C)**	ERR10968291	7.23e+08	109.2
**Sequel IIe (PacBio)**	ERR10962207	5.90e+05	7.63
**Sequel IIe (PacBio)**	ERR10962206	2.11e+06	25.47

Haplotype 1 and haplotype 2 were combined for manual curation. Assembly errors, including 716 missing joins or mis-joins and 5 haplotypic duplications were corrected during curation. The result was to increase the assembly length by 1.68% and the scaffold number by 0.9%, and to increase the scaffold N50 by 2.01%.

**Figure 2.**
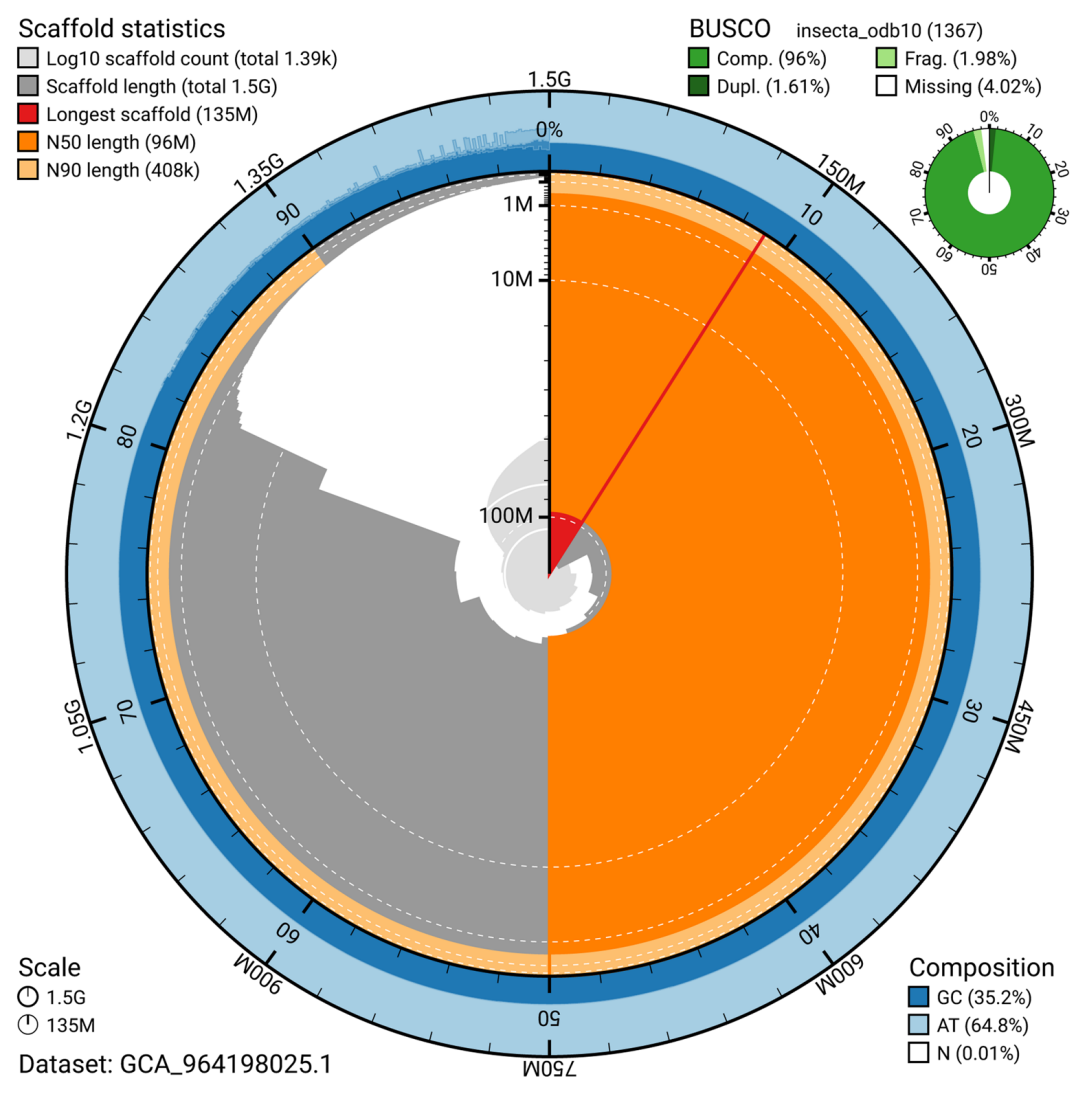
Genome assembly of *Sympetrum sanguineum*, ioSymSang1.hap1.1: metrics. The BlobToolKit snail plot provides an overview of assembly metrics and BUSCO gene completeness. The circumference represents the length of the whole genome sequence, and the main plot is divided into 1,000 bins around the circumference. The outermost blue tracks display the distribution of GC, AT, and N percentages across the bins. Scaffolds are arranged clockwise from longest to shortest and are depicted in dark grey. The longest scaffold is indicated by the red arc, and the deeper orange and pale orange arcs represent the N50 and N90 lengths. A light grey spiral at the centre shows the cumulative scaffold count on a logarithmic scale. A summary of complete, fragmented, duplicated, and missing BUSCO genes in the insecta_odb10 set is presented at the top right. An interactive version of this figure is available at https://blobtoolkit.genomehubs.org/view/Sympetrum_sanguineum/dataset/GCA_964198025.1/snail.

**Figure 3.**
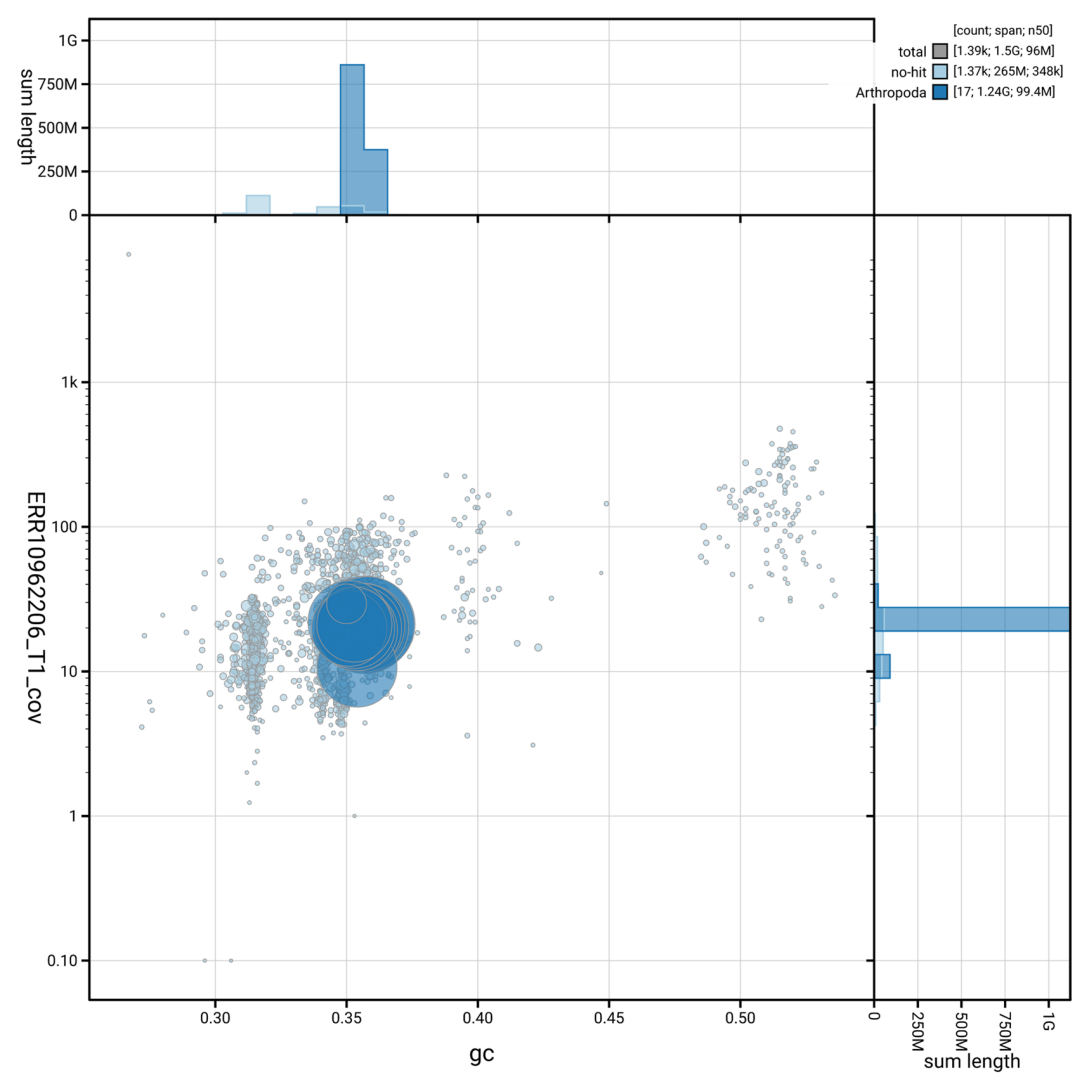
Genome assembly of *Sympetrum sanguineum*, ioSymSang1.hap1.1: BlobToolKit GC-coverage plot. Blob plot showing sequence coverage (vertical axis) and GC content (horizontal axis). The circles represent scaffolds, with the size proportional to scaffold length and the colour representing phylum membership. The histograms along the axes display the total length of sequences distributed across different levels of coverage and GC content. An interactive version of this figure is available at https://blobtoolkit.genomehubs.org/view/Sympetrum_sanguineum/dataset/GCA_964198025.1/blob.

**Figure 4.**
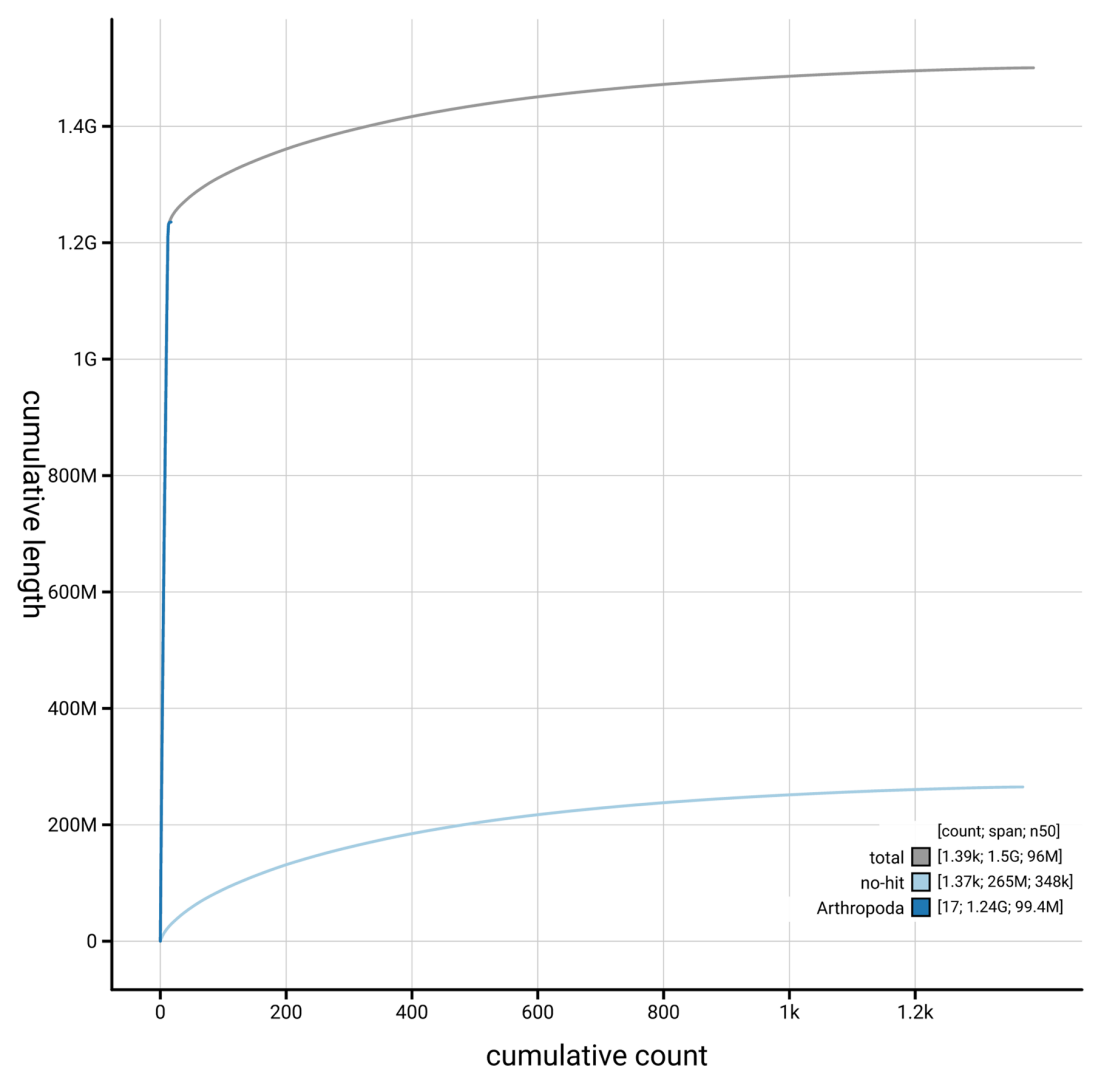
Genome assembly of *Sympetrum sanguineum* ioSymSang1.hap1.1: BlobToolKit cumulative sequence plot. The grey line shows cumulative length for all scaffolds. Coloured lines show cumulative lengths of scaffolds assigned to each phylum using the buscogenes taxrule. An interactive version of this figure is available at https://blobtoolkit.genomehubs.org/view/Sympetrum_sanguineum/dataset/GCA_964198025.1/cumulative.

The final haplotype 1 assembly has a total length of 1,500.53 Mb in 1,387 sequence scaffolds, with 628 gaps, and a scaffold N50 of 96.0 Mb ( Table 2). The snail plot in Figure 2 provides a summary of the assembly statistics for haplotype 1, while the distribution of assembly scaffolds on GC proportion and coverage is shown in Figure 3. The cumulative assembly plot in Figure 4 shows curves for subsets of scaffolds assigned to different phyla.

**Table 2.** Genome assembly data for *Sympetrum sanguineum*, both haplotypes.

Genome assembly	Haplotype 1	Haplotype 2
Assembly name	ioSymSang1.hap1.1	ioSymSang1.hap2.1
Assembly accession	GCA_964198025.1	GCA_964198035.1
Assembly level	chromosome	chromosome
Span (Mb)	1,500.53	1,304.05
Number of contigs	2,015	1,242
Number of scaffolds	1,387	672
Longest scaffold (Mb)	135.43	144.71
Assembly metrics (benchmark) *	Haplotype 1	Haplotype 2
Contig N50 length (≥ 1 Mb)	2.52 Mb	2.62 Mb
Scaffold N50 length (= chromosome N50)	96.0 Mb	96.02 Mb
Consensus quality (QV) (≥ 40)	62.7	63.5
*k*-mer completeness (≥ 95%)	80.59% (combined 98.66%)	75.16%
BUSCO ** (S > 90%; D < 5%)	C:95.9%[S:94.3%,D:1.6%], F:2.0%,M:2.1%,n:1,367	C:91.6%[S:90.3%,D:1.3%], F:2.2%,M:6.2%,n:1,367
Percentage of assembly mapped to chromosomes (≥ 90%)	82.06%	86.66%
Sex chromosomes (localised homologous pairs	X	-
Organelles (one complete allele)	Mitochondrial genome: 17.27 kb	

* Assembly metric benchmarks are adapted from Rhie *et al.* (2021) and the Earth BioGenome Project Report on Assembly Standards September 2024. ** BUSCO scores based on the insecta_odb10 BUSCO set using version 5.4.3. C = complete [S = single copy, D = duplicated], F = fragmented, M = missing, n = number of orthologues in comparison.

**Figure 5.**
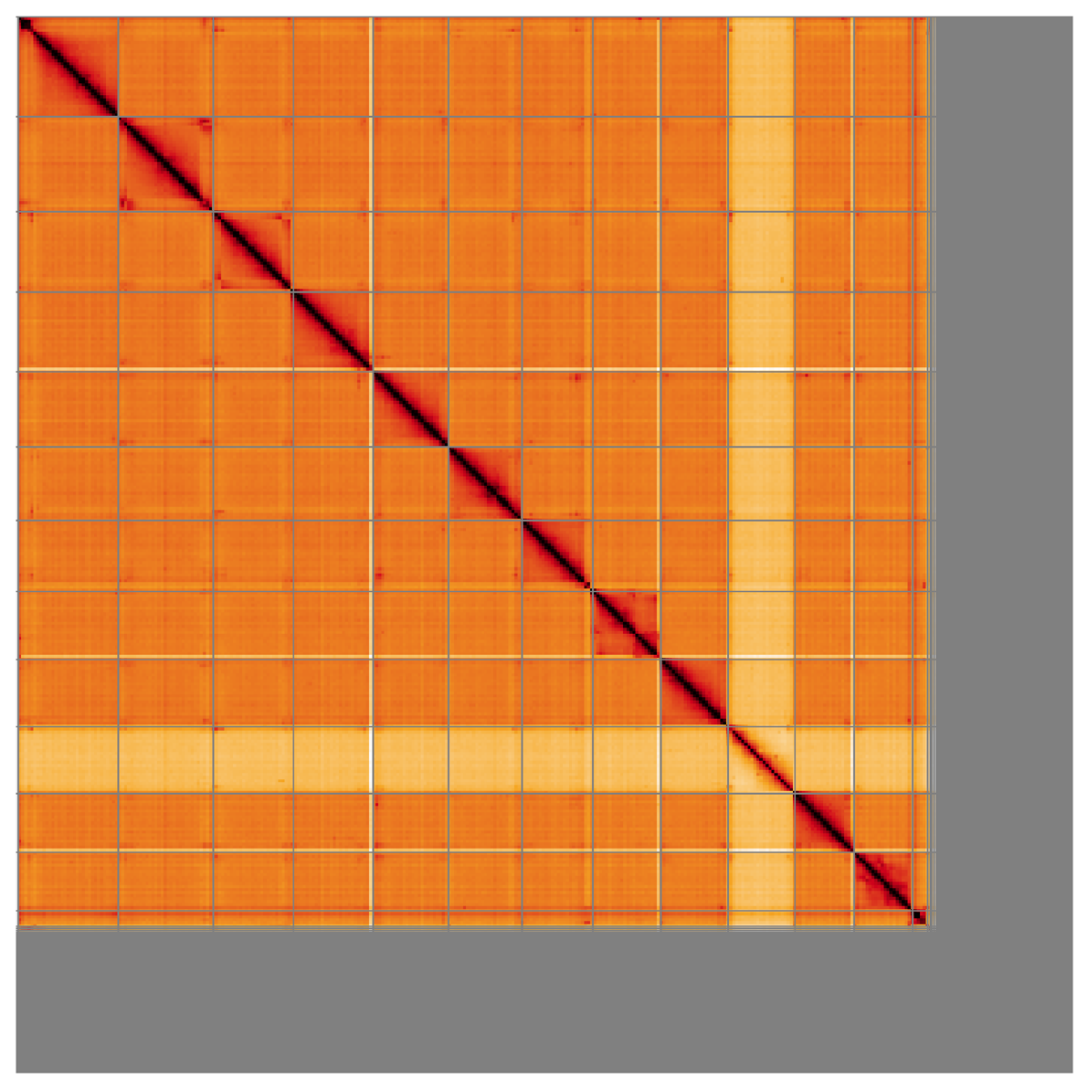
Genome assembly of *Sympetrum sanguineum* ioSymSang1.hap1.1: Hi-C contact map of the ioSymSang1.hap1.1 assembly, visualised using HiGlass. Chromosomes are shown in order of size from left to right and top to bottom. An interactive version of this figure may be viewed at https://genome-note-higlass.tol.sanger.ac.uk/l/?d=KYHOGRoOSn2EckXqEOT1-Q.

For haplotype 1, most (83.39%) of the assembly sequence was assigned to 13 chromosomal-level scaffolds, representing 12 autosomes and the X sex chromosome. The specimen is an XO male. Chromosome-scale scaffolds confirmed by the Hi-C data are named in order of size ( Figure 5; Table 3). During manual curation, haplotypic inversions were identified on Chromosome 2 from 113–128.1 Mb, Chromosome 3 from 95–108.5 Mb, Chromosome 6 from 55.7–63 Mb, Chromosome 8 from 9.8–60.7 Mb and Chromosome 11 from 16.60–38.26 Mb. The order and orientation may not be certain for highly repetitive regions identified on Chromosome 1 from 0 to 14 Mb and Chromosome 7 from 86.5 to 95.9 Mb. The mitochondrial genome was also assembled and is included both as a contig within the multifasta file of the genome submission and as a standalone record in GenBank.

**Table 3.** Chromosomal pseudomolecules in the genome assembly of *Sympetrum sanguineum*, ioSymSang1.

Haplotype 1				Haplotype 2			
INSDC accession	Name	Length (Mb)	GC%	INSDC accession	Name	Length (Mb)	GC%
OZ078875.1	1	135.43	35.5	OZ078863.1	1	144.71	35.5
OZ078876.1	2	128.59	36	OZ078864.1	2	129.28	36
OZ078877.1	3	108.6	35.5	OZ078865.1	3	104.81	35.5
OZ078878.1	4	108.06	35.5	OZ078866.1	4	105.49	35.5
OZ078879.1	5	101.93	35.5	OZ078867.1	5	104.18	35.5
OZ078880.1	6	99.38	35.5	OZ078868.1	6	96.02	35.5
OZ078881.1	7	96.0	35	OZ078871.1	7	85.92	35.5
OZ078883.1	8	92.25	35.5	OZ078869.1	8	93.44	35.5
OZ078884.1	9	90.99	35.5	OZ078870.1	9	90.72	35.5
OZ078885.1	10	79.96	35	OZ078872.1	10	80.82	35
OZ078886.1	11	79.07	35.5	OZ078873.1	11	74.63	35
OZ078887.1	12	20.64	35	OZ078874.1	12	20.09	35
OZ078882.1	X	90.43	35.5				
OZ078888.1	MT	0.02	26.5				

The estimated Quality Value (QV) of the haplotype 1 assembly is 62.7 with *k*-mer completeness of 80.59%, and the assembly has a BUSCO v5.4.3 completeness of 95.9% (single = 94.3%, duplicated = 1.6%), using the insecta_odb10 reference set ( *n* = 1,367).

For haplotype 2, the estimated Quality Value (QV) of the final assembly is 63.5 with *k*-mer completeness of 75.16%. The assembly has a BUSCO v5.4.3 completeness of 91.6% (single = 90.3%, duplicated = 1.3%), using the insecta_odb10 reference set ( *n* = 1,367). The *k*-mer completeness of the combined assemblies is 98.66%.

Metadata for specimens, BOLD barcode results, spectra estimates, sequencing runs, contaminants and pre-curation assembly statistics are given at https://links.tol.sanger.ac.uk/species/197176.

## Methods

### Sample acquisition and DNA barcoding

An adult male *Sympetrum sanguineum* (specimen ID Ox001974, ToLID ioSymSang1) was collected from Wytham Woods, Berkshire, United Kingdom (latitude 51.76, longitude –1.34) on 2021-10-10 by netting. The specimen was collected and identified by Liam Crowley (University of Oxford) and preserved on dry ice.

The initial identification was verified by an additional DNA barcoding process according to the framework developed by Twyford *et al*. (2024). A small sample was dissected from the specimens and stored in ethanol, while the remaining parts were shipped on dry ice to the Wellcome Sanger Institute (WSI). The tissue was lysed, the COI marker region was amplified by PCR, and amplicons were sequenced and compared to the BOLD database, confirming the species identification ( Crowley *et al.*, 2023). Following whole genome sequence generation, the relevant DNA barcode region was also used alongside the initial barcoding data for sample tracking at the WSI ( Twyford *et al.*, 2024). The standard operating procedures for Darwin Tree of Life barcoding have been deposited on protocols.io ( Beasley *et al.*, 2023).

### Nucleic acid extraction

The workflow for high molecular weight (HMW) DNA extraction at the Wellcome Sanger Institute (WSI) Tree of Life Core Laboratory includes a sequence of core procedures: sample preparation and homogenisation, DNA extraction, fragmentation and purification. Detailed protocols are available on protocols.io ( Denton *et al.*, 2023b). The ioSymSang1 sample was prepared for DNA extraction by weighing and dissecting it on dry ice ( Jay *et al.*, 2023), and tissue from the thorax was homogenised using a PowerMasher II tissue disruptor ( Denton *et al.*, 2023a).

HMW DNA was extracted in the WSI Scientific Operations core using the Automated MagAttract v2 protocol ( Oatley *et al.*, 2023). The DNA was sheared into an average fragment size of 12–20 kb in a Megaruptor 3 system ( Bates *et al.*, 2023). Sheared DNA was purified by solid-phase reversible immobilisation, using AMPure PB beads to eliminate shorter fragments and concentrate the DNA ( Strickland *et al.*, 2023). The concentration of the sheared and purified DNA was assessed using a Nanodrop spectrophotometer and Qubit Fluorometer using the Qubit dsDNA High Sensitivity Assay kit. Fragment size distribution was evaluated by running the sample on the FemtoPulse system.

### Hi-C preparation

Tissue from the thorax of the ioSymSang1 sample was processed at the WSI Scientific Operations core, using the Arima-HiC v2 kit. Frozen tissue (stored at –80 °C) was fixed, and the DNA crosslinked using a TC buffer with 22% formaldehyde. After crosslinking, the tissue was homogenised using the Diagnocine Power Masher-II and BioMasher-II tubes and pestles. Following the kit manufacturer's instructions, crosslinked DNA was digested using a restriction enzyme master mix. The 5’-overhangs were then filled in and labelled with biotinylated nucleotides and proximally ligated. An overnight incubation was carried out for enzymes to digest remaining proteins and for crosslinks to reverse. A clean up was performed with SPRIselect beads prior to library preparation.

### Library preparation and sequencing

Library preparation and sequencing were performed at the WSI Scientific Operations core. Pacific Biosciences HiFi circular consensus DNA sequencing libraries were prepared using the PacBio Express Template Preparation Kit v2.0 (Pacific Biosciences, California, USA) as per the manufacturer’s instructions. The kit includes the reagents required for removal of single-strand overhangs, DNA damage repair, end repair/A-tailing, adapter ligation, and nuclease treatment. Library preparation also included a library purification step using AMPure PB beads (Pacific Biosciences, California, USA) and size selection step to remove templates shorter than 3 kb using AMPure PB modified SPRI. DNA concentration was quantified using the Qubit Fluorometer v2.0 and Qubit HS Assay Kit and the final library fragment size analysis was carried out using the Agilent Femto Pulse Automated Pulsed Field CE Instrument and 165kb gDNA and 55kb BAC analysis kit. Samples were sequenced using the Sequel IIe system (Pacific Biosciences, California, USA). The concentration of the library loaded onto the Sequel IIe was between 40–135 pM. The SMRT link software, a PacBio web-based end-to-end workflow manager, was used to set-up and monitor the run, as well as perform primary and secondary analysis of the data upon completion.

For Hi-C library preparation, DNA was fragmented to a size of 400 to 600 bp using a Covaris E220 sonicator. The DNA was then enriched, barcoded, and amplified using the NEBNext Ultra II DNA Library Prep Kit following manufacturers’ instructions. The Hi-C sequencing was performed using paired-end sequencing with a read length of 150 bp on an Illumina NovaSeq 6000 instrument.

### Genome assembly, curation and evaluation

***Assembly***

The HiFi reads were first assembled using Hifiasm ( Cheng *et al.*, 2021; Cheng *et al.*, 2022) in Hi-C phasing mode, resulting in a pair of haplotype-resolved assemblies. The Hi-C reads were mapped to the primary contigs using bwa-mem2 ( Vasimuddin *et al.*, 2019). The contigs were further scaffolded using the provided Hi-C data ( Rao *et al.*, 2014) in YaHS ( Zhou *et al.*, 2023) using the --break option for handling potential misassemblies. The scaffolded assemblies were evaluated using Gfastats ( Formenti *et al.*, 2022), BUSCO ( Manni *et al.*, 2021) and MERQURY.FK ( Rhie *et al.*, 2020).

The mitochondrial genome was assembled using MitoHiFi ( Uliano-Silva *et al.*, 2023), which runs MitoFinder ( Allio *et al.*, 2020) and uses these annotations to select the final mitochondrial contig and to ensure the general quality of the sequence.

***Assembly curation***

The assembly was decontaminated using the Assembly Screen for Cobionts and Contaminants (ASCC) pipeline (article in preparation). Flat files and maps used in curation were generated in TreeVal ( Pointon *et al.*, 2023). Manual curation was primarily conducted using PretextView ( Harry, 2022), with additional insights provided by JBrowse2 ( Diesh *et al.*, 2023) and HiGlass ( Kerpedjiev *et al.*, 2018). Scaffolds were visually inspected and corrected as described by Howe *et al*. (2021). Any identified contamination, missed joins, and mis-joins were corrected, and duplicate sequences were tagged and removed. The curation process is documented at https://gitlab.com/wtsi-grit/rapid-curation (article in preparation).

***Evaluation of the final assembly***

The final assembly was post-processed and evaluated using the three Nextflow ( Di Tommaso *et al.*, 2017) DSL2 pipelines: sanger-tol/readmapping ( Surana *et al.*, 2023a), sanger-tol/genomenote ( Surana *et al.*, 2023b), and sanger-tol/blobtoolkit ( Muffato *et al.*, 2024). The readmapping pipeline aligns the Hi-C reads using bwa-mem2 ( Vasimuddin *et al.*, 2019) and combines the alignment files with SAMtools ( Danecek *et al.*, 2021). The genomenote pipeline converts the Hi-C alignments into a contact map using BEDTools ( Quinlan & Hall, 2010) and the Cooler tool suite ( Abdennur & Mirny, 2020). The contact map is visualised in HiGlass ( Kerpedjiev *et al.*, 2018). This pipeline computes *k*-mer completeness and QV consensus quality values with FastK and MERQURY.FK, and runs BUSCO ( Manni *et al.*, 2021) to assess completeness.

The blobtoolkit pipeline is a Nextflow port of the previous Snakemake Blobtoolkit pipeline ( Challis *et al.*, 2020). It aligns the PacBio reads in SAMtools and minimap2 ( Li, 2018) and generates coverage tracks for regions of fixed size. In parallel, it queries the GoaT database ( Challis *et al.*, 2023) to identify all matching BUSCO lineages to run BUSCO ( Manni *et al.*, 2021). For the three domain-level BUSCO lineages, the pipeline aligns the BUSCO genes to the UniProt Reference Proteomes database ( Bateman *et al.*, 2023) with DIAMOND ( Buchfink *et al.*, 2021) blastp. The genome is also split into chunks according to the density of the BUSCO genes from the closest taxonomic lineage, and each chunk is aligned to the UniProt Reference Proteomes database with DIAMOND blastx. Genome sequences without a hit are chunked with seqtk and aligned to the NT database with blastn ( Altschul *et al.*, 1990). The blobtools suite combines all these outputs into a blobdir for visualisation.

The genome evaluation pipelines were developed using nf-core tooling ( Ewels *et al.*, 2020) and MultiQC ( Ewels *et al.*, 2016), relying on the Conda package manager, the Bioconda initiative ( Grüning *et al.*, 2018), the Biocontainers infrastructure ( da Veiga Leprevost *et al.*, 2017), as well as the Docker ( Merkel, 2014) and Singularity ( Kurtzer *et al.*, 2017) containerisation solutions.

Table 4 contains a list of relevant software tool versions and sources.

**Table 4.** Software tools: versions and sources.

Software tool	Version	Source
BEDTools	2.30.0	https://github.com/arq5x/bedtools2
BLAST	2.14.0	ftp://ftp.ncbi.nlm.nih.gov/blast/executables/blast+/
BlobToolKit	4.3.7	https://github.com/blobtoolkit/blobtoolkit
BUSCO	5.4.3 and 5.5.0	https://gitlab.com/ezlab/busco
bwa-mem2	2.2.1	https://github.com/bwa-mem2/bwa-mem2
Cooler	0.8.11	https://github.com/open2c/cooler
DIAMOND	2.1.8	https://github.com/bbuchfink/diamond
fasta_windows	0.2.4	https://github.com/tolkit/fasta_windows
FastK	427104ea91c78c3b8b8b49f1a7d6bbeaa869ba1c	https://github.com/thegenemyers/FASTK
Gfastats	1.3.6	https://github.com/vgl-hub/gfastats
GoaT CLI	0.2.5	https://github.com/genomehubs/goat-cli
Hifiasm	0.19.8-r603	https://github.com/chhylp123/hifiasm
HiGlass	44086069ee7d4d3f6f3f0012569789ec138f42b84 aa44357826c0b6753eb28de	https://github.com/higlass/higlass
Merqury.FK	d00d98157618f4e8d1a9190026b19b471055b22e	https://github.com/thegenemyers/MERQURY.FK
MitoHiFi	3	https://github.com/marcelauliano/MitoHiFi
MultiQC	1.14, 1.17, and 1.18	https://github.com/MultiQC/MultiQC
NCBI Datasets	15.12.0	https://github.com/ncbi/datasets
Nextflow	23.04.0-5857	https://github.com/nextflow-io/nextflow
PretextView	0.2	https://github.com/sanger-tol/PretextView
purge_dups	1.2.5	https://github.com/dfguan/purge_dups
samtools	1.16.1, 1.17, and 1.18	https://github.com/samtools/samtools
sanger-tol/ascc	-	https://github.com/sanger-tol/ascc
sanger-tol/genomenote	1.1.1	https://github.com/sanger-tol/genomenote
sanger-tol/readmapping	1.2.1	https://github.com/sanger-tol/readmapping
Seqtk	1.3	https://github.com/lh3/seqtk
Singularity	3.9.0	https://github.com/sylabs/singularity
TreeVal	1.0.0	https://github.com/sanger-tol/treeval
YaHS	1.2a.2	https://github.com/c-zhou/yahs

### Wellcome Sanger Institute – Legal and Governance

The materials that have contributed to this genome note have been supplied by a Darwin Tree of Life Partner. The submission of materials by a Darwin Tree of Life Partner is subject to the **‘Darwin Tree of Life Project Sampling Code of Practice’**, which can be found in full on the Darwin Tree of Life website here. By agreeing with and signing up to the Sampling Code of Practice, the Darwin Tree of Life Partner agrees they will meet the legal and ethical requirements and standards set out within this document in respect of all samples acquired for, and supplied to, the Darwin Tree of Life Project.

Further, the Wellcome Sanger Institute employs a process whereby due diligence is carried out proportionate to the nature of the materials themselves, and the circumstances under which they have been/are to be collected and provided for use. The purpose of this is to address and mitigate any potential legal and/or ethical implications of receipt and use of the materials as part of the research project, and to ensure that in doing so we align with best practice wherever possible. The overarching areas of consideration are:

•   Ethical review of provenance and sourcing of the material

•   Legality of collection, transfer and use (national and international)

Each transfer of samples is further undertaken according to a Research Collaboration Agreement or Material Transfer Agreement entered into by the Darwin Tree of Life Partner, Genome Research Limited (operating as the Wellcome Sanger Institute), and in some circumstances other Darwin Tree of Life collaborators.

## Data Availability

European Nucleotide Archive: Sympetrum sanguineum (ruddy darter). Accession number PRJEB60309; https://identifiers.org/ena.embl/PRJEB60309. The genome sequence is released openly for reuse. The *Sympetrum sanguineum* genome sequencing initiative is part of the Darwin Tree of Life (DToL) project. All raw sequence data and the assembly have been deposited in INSDC databases. The genome will be annotated using available RNA-Seq data and presented through the Ensembl pipeline at the European Bioinformatics Institute. Raw data and assembly accession identifiers are reported in Table 1 and Table 2.

## Author information

Members of the University of Oxford and Wytham Woods Genome Acquisition Lab are listed here: https://doi.org/10.5281/zenodo.12157525.

Members of the Darwin Tree of Life Barcoding collective are listed here: https://doi.org/10.5281/zenodo.12158331.

Members of the Wellcome Sanger Institute Tree of Life Management, Samples and Laboratory team are listed here: https://doi.org/10.5281/zenodo.12162482.

Members of Wellcome Sanger Institute Scientific Operations: Sequencing Operations are listed here: https://doi.org/10.5281/zenodo.12165051.

Members of the Wellcome Sanger Institute Tree of Life Core Informatics team are listed here: https://doi.org/10.5281/zenodo.12160324.

Members of the Tree of Life Core Informatics collective are listed here: https://doi.org/10.5281/zenodo.12205391.

Members of the Darwin Tree of Life Consortium are listed here: https://doi.org/10.5281/zenodo.4783558.
