# Supplementary material for: The genome sequence of the Ruddy Darter, Sympetrum sanguineum (Müller, 1764)
Source: Wellcome Open Res. 2025 Jan 15;10:23. [Version 1] doi: 10.12688/wellcomeopenres.23466.1 (PMC11868746; doi:10.12688/wellcomeopenres.23466.1)
Supplement: Peer review history

# Reviewer report: Reviewer response for version 1

**DOI:** 10.21956/wellcomeopenres.25876.r118224

**Published:** 2025-03-25

**Authors:** Cinta Pegueroles, Ainhoa López

**Affiliations:** 1 Universitat Autonoma de Barcelona, Barcelona, Catalonia, Spain; 2 Genètica, Microbiologia i Estadística, Universitat de Barcelona Departament de Genetica Microbiologia i Estadistica (Ringgold ID: 200148), Barcelona, Catalonia, Spain

The authors present the first genome assembly of the Ruddy Darter dragonfly ( *Sympetrum sanguineum*). It is a high-quality assembly resolved at both haplotype and chromosomal levels. We appreciate that all generated data is publicly available in the ENA repository. Quality statistics are overall good, highlighting the importance of this new genome resource. However, there are some parts of the article that need some revision. Some methods are difficult to follow, especially since they are based on unpublished works. Below, we indicate the parts that should be rewritten for clarity. Finally, it is unfortunate that the genome has not been annotated yet, and we strongly encourage doing so in the near future.

 Here we detail our suggestions to improve the manuscript.

 As mentioned above, some methods are difficult to follow. In the Assembly curation section, two processes are cited with an “article in preparation”, the decomposition, and the final curation, which difficult their evaluation. Because this section is part of the methodology, it could be better to have the reference, even if the paper is not peer-reviewed, such as a bioarxiv paper. 

 Regarding methods, how were the haplotypic duplications detected?

 Regarding manual curation, in the main text it is stated  “Haplotype 1 and haplotype 2 were combined for manual curation”. If so, why is the combined genome assembly not provided in ENA? Were the two haplotypes manually curated separately? 

 In the background section, there is a lack of citations when describing the species. In addition, it is not clear how the flight information can be linked with the newly generated reference genome. We suggest adding more bibliography and references information about the current genetic knowledge on the species and how the new generated genome will benefit future genetic studies. 

 In the abstract it appears: “Most of haplotype 1 is scaffolded into 13 chromosomal pseudomolecules, including the X sex chromosome, while haplotype 2 is scaffolded into 12 autosomes.” -> Since “chromosomal pseudomolecules” and “autosomes” refer to the same thing, and should be rewritten for clarity.

 How was the sex of the reference individual identified?

 We understand that repeat elements were not identified in this manuscript, since genome annotation is not addressed at all. However, detecting repeat elements for instance using RepeatMasker software is fast and straightforward, being a nice addition to the present manuscript and future studies.

 Ainhoa Lopez and Cinta Pegueroles

Are sufficient details of methods and materials provided to allow replication by others?

Partly

Is the rationale for creating the dataset(s) clearly described?

Partly

Are the datasets clearly presented in a useable and accessible format?

Yes

Are the protocols appropriate and is the work technically sound?

Yes

Reviewer Expertise:

Ainhoa López: genome assembly and annotation, population genomics, bioinformatics. Cinta Pegueroles: genome assembly and annotation, population genomics, bioinformatics.

We confirm that we have read this submission and believe that we have an appropriate level of expertise to confirm that it is of an acceptable scientific standard, however we have significant reservations, as outlined above.

# Reviewer report: Reviewer response for version 1

**DOI:** 10.21956/wellcomeopenres.25876.r118222

**Published:** 2025-02-27

**Authors:** Andrea Galimberti

**Affiliations:** 1 University of Milano-Bicocca, Milan, Italy

This study illustrates the methodological aspects used to assemble the genome of a very common anisopteran species in Europe and of ecological interest.

 The work carried out is very important for filling some gaps in the genetic (and especially genomic) knowledge of European odonates, which are widely used as bio-indicators but not yet sufficiently understood on a multi-omic level.

 Having read the manuscript carefully, I have only a few suggestions to bring to the authors' attention:

 - I would suggest that regarding morphology, distribution and ecology of this species, the authors could also refer to the excellent, more updated, and widely used reference: Dijkstra, K. D., & Schröter, A. (2020). *Field guide to the dragonflies of Britain and Europe*. Bloomsbury Publishing.

 - It would be nice to include a brief overview of genetic studies (from DNA barcoding to genome-scale studies) that have involved this species, especially if they include British populations. It should also be of interest to verify which portion of the overall species range has been covered by genetic surveys at any title.

 - Concerning Figure 2: what are the light blue bars appearing between 80-100%? Are these mentioned in the image caption?

Are sufficient details of methods and materials provided to allow replication by others?

Yes

Is the rationale for creating the dataset(s) clearly described?

Yes

Are the datasets clearly presented in a useable and accessible format?

Yes

Are the protocols appropriate and is the work technically sound?

Yes

Reviewer Expertise:

Molecular taxonomy, Odonates, eDNA-based tools

I confirm that I have read this submission and believe that I have an appropriate level of expertise to confirm that it is of an acceptable scientific standard.

## References

[1] Dijkstra, K. D., & Schröter, A. (2020). Field guide to the dragonflies of Britain and Europe. Bloomsbury Publishing.

# Reviewer report: Reviewer response for version 1

**DOI:** 10.21956/wellcomeopenres.25876.r118227

**Published:** 2025-02-10

**Authors:** Jason Charamis

**Affiliations:** 1 Foundation for Research and Technology - Hellas, Irákleion, Greece

This genome sequencing study of the Ruddy Darter dragonfly ( *Sympetrum sanguineum*) represents an important contribution to our understanding of dragonfly genetics. Given the current scarcity of genomic data for both dragonflies and Paleoptera more broadly, this work provides a valuable resource for researchers exploring evolutionary relationships and ecological adaptations within these groups. The methodology is robust and the manuscript is well-structured. However, I suggest two additions that would strengthen the paper:

 1. The section describing the species' anatomy and ecology would benefit from additional citations, particularly for key claims about population dynamics. For instance, the assertion that "migrants from Continental Europe boosting the population" in the second paragraph requires supporting evidence from the scientific literature.

 2. I recommend expanding the discussion of why sequencing Paleoptera species is significant. The authors could elaborate on how this genomic data enables more comprehensive comparative genomics studies between primitive and more recently evolved insect groups, potentially revealing important insights about insect evolution.

Are sufficient details of methods and materials provided to allow replication by others?

Yes

Is the rationale for creating the dataset(s) clearly described?

No

Are the datasets clearly presented in a useable and accessible format?

Yes

Are the protocols appropriate and is the work technically sound?

Yes

Reviewer Expertise:

Arthropod Comparative Genomics

I confirm that I have read this submission and believe that I have an appropriate level of expertise to confirm that it is of an acceptable scientific standard.
